# Isolation and characterization of avian metapneumovirus subtypes A and B associated with the 2024 disease outbreaks among poultry in the USA

**DOI:** 10.1128/jcm.00333-25

**Published:** 2025-07-10

**Authors:** Jianqiang Zhang, Liying Tian, Jaime Dittman, Baoqing Guo, Kay Kimpston-Burkgren, Erin Kalkwarf, Eman Gadu, Phillip Gauger, Mohamed El-Gazzar, Yuko Sato

**Affiliations:** 1 Veterinary Diagnostic and Production Animal Medicine, College of Veterinary Medicine, Iowa State University 70724 , Ames, Iowa, USA; 2 Department of Avian and Rabbit Diseases, Faculty of Veterinary Medicine, Mansoura University 158400 , Mansoura, Egypt; University of California, Davis, Davis, California, USA

**Keywords:** avian metapneumovirus, aMPV, subtype, virus isolation, primary chicken embryo fibroblast cells, primary chicken embryo lung cells, Vero cell line, whole genome sequencing

## Abstract

Avian metapneumovirus (aMPV) is currently classified into four subtypes (aMPV-A, -B, -C, and -D). In late 2023 and early 2024, aMPV-A and aMPV-B were detected in US poultry for the first time, causing significant economic losses. This study analyzed aMPV RT-PCR data from 2,204 samples (1,158 turkey, 936 chicken, and 110 other breeds) submitted to a US veterinary diagnostic laboratory between January and November 2024. A higher percentage of turkey samples (51.04%) tested PCR-positive for aMPV-A and/or aMPV-B compared to chicken samples (15.6%), with aMPV-A showing an overall higher positive rate than aMPV-B, although the positive rates varied by state. Additionally, four aMPV-A and three aMPV-B isolates were successfully recovered from clinical samples using primary chicken embryo lung and/or fibroblast cells. Two aMPV-A isolates (USA/IA55601-6/2024 and USA/IA56509-5/2024) and two aMPV-B isolates (USA/NC20487-GA/2024 and USA/NC23734-GA/2024) were adapted to grow efficiently in a Vero cell line, reaching titers of ~10^4^–10^6^ TCID_50_/mL between passages 4 and 10 for aMPV-A and between passages 1 and 10 for aMPV-B. Whole genome sequencing of the two aMPV-A and two aMPV-B isolates at different passages revealed that the viruses progressively acquired several nucleotide changes, some of which led to amino acid substitutions in different viral proteins during 10 passages in cell culture. Comparative analysis with 46 aMPV-A, -B, -C, and -D GenBank sequences showed that US aMPV-A and aMPV-B strains were genetically closely related within their subtypes. These cell culture-adapted US aMPV-A and aMPV-B isolates provide valuable tools for further characterization of aMPV and vaccine development.

**IMPORTANCE:**

Avian metapneumovirus (aMPV) subtypes A and B were first detected in US poultry in late 2023 and early 2024, rapidly spreading nationwide and posing a significant threat to the industry. This study analyzed RT-PCR data from 2,204 clinical samples (January to November 2024) to determine aMPV-A and aMPV-B detection rates across poultry species, age groups, and states, providing insights into their epidemiology in the USA. Modified live vaccines are urgently needed to control aMPV but are hindered by the lack of US isolates growing efficiently in cell culture. We successfully isolated aMPV-A and aMPV-B in primary chicken embryo cells and adapted them to a Vero cell line. Their infectious titers and genetic stability were characterized over serial passages. These US aMPV-A and aMPV-B cell culture isolates provide valuable tools for studying pathogenesis, determining virus infectious doses, evaluating disinfectants and antivirals, and developing vaccines.

## INTRODUCTION

Avian metapneumovirus (aMPV), previously referred to as avian pneumovirus or avian rhinotracheitis virus or turkey rhinotracheitis virus, can infect various bird species. However, it primarily affects turkeys and chickens, causing respiratory tract infections characterized by sinusitis, swollen heads, increased mortality, and reduced egg production, leading to significant economic losses (1). Co-infection with aMPV and other organisms, including bacteria, may lead to a more severe form of the disease (2, 3).

Avian metapneumovirus is a member of the genus *Metapneumovirus* in the family *Pneumoviridae*. The aMPV comprises a single-stranded, non-segmented, and negative-sense RNA genome with a length of ~13.3–14 kb, which contains nine genes arranged in the order of 3′-N–P–M–F–M2.1–M2.2–SH–G–L-5′ with intergenic regions between every two adjacent genes; in addition, the genome includes untranslated regions (UTRs) on both the 3´ and 5´ ends (4). The corresponding proteins encoded by these genes are nucleoprotein (N), phosphoprotein (P), matrix protein (M), fusion protein (F), second matrix proteins (M2.1 and M2.2), small hydrophobic protein (SH), surface glycoprotein (G), and large polymerase protein (L). aMPV is currently classified into four subtypes (aMPV-A, -B, -C, and -D) (5), but two new unclassified subtypes have recently been discovered in a Monk parakeet and a great black-backed gull (6, 7).

The first detection of aMPV occurred in turkeys in South Africa in 1978, and it was later determined to be aMPV-A (5). Subsequently, aMPV-A was detected in the UK, France, and other European countries (8). aMPV-A was also reported in South Korea in 2010 (9). Recently, aMPV-A has been detected in Mexico and Morocco (10, 11). aMPV-B was first detected in Europe during the 1980s and subsequently established endemic infections in numerous European countries (8, 12, 13). In the past few years, aMPV-B has been detected in multiple continents, such as Morocco and Tunisia in Africa (11, 14), South Korea, China, and Iraq in Asia (15, 16), and Brazil and Colombia in South America (17, 18). aMPV-C was first identified in turkeys in the USA in 1996 (19). Subsequently, aMPV-C was detected in numerous countries, such as Canada, China, South Korea, France, Netherlands, Italy, and so on (20–26). aMPV-D was retrospectively identified in archival samples from turkeys in France in 1985 (27), but it has not been reported since.

After the first detection in Colorado, USA, in 1996, aMPV-C became endemic in multiple states of the USA during the late 1990s and early 2000s. However, after the implementation of live attenuated vaccines together with outbreak tracing and containment measures, aMPV-C has not been detected in poultry or wildlife in the USA for over a decade (28). aMPV-A and aMPV-B had not previously been identified in the USA, but the status changed recently. The emergence of aMPV-A in California and aMPV-B in North Carolina and Virginia in turkeys and broilers was reported for the first time in the USA in late 2023 and early 2024, followed by rapid spread of the viruses to most poultry-producing regions nationwide (28–30). According to the US Animal Health Association Turkey Industry survey released in October 2024, aMPV was ranked the number 1 disease threat (31). While enhancing management practices and biosecurity measures are essential for controlling and preventing the spread of aMPV, vaccination remains a critical tool for minimizing illness and economic losses, especially by priming the immunity with a live vaccine and boosting the immunity with an inactivated vaccine. Three experimental autogenous vaccines, with two containing inactivated aMPV-B strain of the US origin (manufactured by Merck Animal Health/Cambridge Technologies, USA and Ceva, USA, respectively) and one containing inactivated aMPV-A strain of the US origin (manufactured by Vaxxinova, USA), have been approved by the United States Department of Agriculture (USDA) for use. However, modified live virus (MLV) vaccines based on aMPV-A or aMPV-B of the US origin are not available yet in the USA. As of 25 March 2025, the USDA has approved the importation of five live aMPV vaccines and two inactivated aMPV vaccines from other countries for emergency use in the USA to control aMPV. These imported vaccines include: live vaccine Vaxxon SHS (aMPV-B, Vaxxinova, Italy), live vaccine NEMOVAC (aMPV-B, Boehringer Ingelheim, France) for use in chickens, live vaccine AVIFFA RTI (aMPV-B, Boehringer Ingelheim, France) for use in turkeys, live vaccine Poulvac TRT (aMPV-A, Zoetis, Spain), live vaccine RESPIVAC aMPV (aMPV-B, HIPRA, Spain), inactivated vaccine HIPRAVIAR TRT (aMPV-B, HIPRA, Spain), and inactivated vaccine TUR-3 (aMPV-B, Boehringer Ingelheim, France). Although these imported aMPV MLV vaccines are approved for emergency use in the USA, their protective efficacy against the US strains has yet to be determined, necessitating the use of US aMPV-A and aMPV-B isolates as challenge viruses. Furthermore, it may still be essential to develop MLV vaccines based on US aMPV-A and aMPV-B strains.

Avian metapneumovirus is notoriously difficult to isolate due to its limited period of viral shedding post-infection and its instability in diagnostic specimens (1, 32). Additionally, aMPV may require critical nucleotide and/or amino acid changes in certain viral proteins during virus isolation (VI) to establish a stably replicating isolate. As a result, very few laboratories have been successful in isolating and growing aMPV-A and aMPV-B in cell culture, especially in continuous cell lines.

In this study, our first objective was to assess the RT-PCR detection frequency of aMPV-A and aMPV-B across different poultry species, age groups, and US states, as such epidemiological data are currently lacking. The second objective was to attempt virus isolation in order to obtain US aMPV-A and aMPV-B isolates and adapt them to grow in a continuous cell line.

## MATERIALS AND METHODS

### Clinical samples submitted to Iowa State University Veterinary Diagnostic Laboratory (ISU VDL) for aMPV RT-PCR testing between January and November 2024

A total of 2,204 clinical samples tested by aMPV-A, aMPV-B, and aMPV-C real-time RT-PCR at ISU VDL from January to November 2024 were included for analysis in this study.

### Nucleic acid extraction

Nucleic acids were extracted from samples using a MagMAX Pathogen RNA/DNA Kit (Thermo Fisher Scientific, Waltham, MA, USA) and a Kingfisher Apex instrument (Thermo Fisher Scientific) following the manufacturer’s instructions. One hundred microliters of the sample was used for extraction, and nucleic acid was eluted into 90 µL elution buffer. Before nucleic acid extraction, an internal positive control XIPC RNA (1 × 10^4^ copies per extraction) was added to the extraction lysis buffer (33). As a result, the extracted nucleic acid from each sample was expected to contain the XIPC RNA in addition to the target pathogen nucleic acid (33). XIPC DNA is a fragment of nucleotides that was artificially designed and synthesized with T7 promoter at the 5′ upstream. The XIPC sequence is not present in any analyzed pathogens or host species. XIPC DNA is *in vitro* transcribed into XIPC RNA, which is routinely used in our laboratory. Primers and probes for XIPC are proprietary products developed in our laboratory, and their sequences are available upon request.

### Avian metapneumovirus subtypes A, B, and C real-time RT-PCR

Previously described aMPV-A, aMPV-B, and aMPV-C real-time RT-PCR assays, targeting the G gene of aMPV-A and aMPV-B and the SH gene of aMPV-C (34), were used in this study to test for the presence of aMPV-A, -B, and -C in clinical samples and cell culture isolates. In brief, each individual RT-PCR was set up in a 20 µL reaction: 5 µL of TaqMan Fast 1-Step Master Mix (Thermo Fisher Scientific), 0.8 µL of forward primer at 10 µM, 0.8 µL of reverse primer at 10 µM, 0.16 µL of probe at 25 µM, 0.2 µL XIPC forward primer at 20 µM, 0.2 µL of XIPC reverse primer at 20 µM, 0.15 µL of XIPC probe at 10 µM, 4.69 µL nuclease-free water, and 8 µL nucleic acid extract. Amplification reactions were performed on an ABI 7500 Fast Instrument (Thermo Fisher Scientific) with the following RT-PCR conditions: one cycle of 50°C for 5 min, one cycle of 95°C for 20 s, and 40 cycles of 95°C for 15 s and 60°C for 30 s. The analysis was performed using an automatic baseline, probe detector at a threshold of 0.1, and an XIPC detector (Cy5) at 10% of the maximum height of the sigmoid amplification curve. Samples with Ct <37 were considered PCR-positive, and samples with Ct ≥37 were considered PCR-negative for aMPV. Historical aMPV subtype A (UK strain), subtype B (Hungary strain), and subtype C (Colorado strain), purchased from the National Veterinary Service Laboratory (NVSL, Ames, IA, USA) of the USDA, were used as positive controls. Phosphate-buffered saline (PBS) and nuclease-free water were used as negative controls.

### Cell lines, embryonated chicken eggs (ECEs), and primary chicken embryo cells

African green monkey kidney cell line Vero (ATCC CCL-81) and chicken embryo fibroblast (CEF) cell line UMNSAH/DF-1 (ATCC CRL-3586) were cultured and maintained in Dulbecco’s Modified Eagle Medium (DMEM, Thermo Fisher Scientific) supplemented with 10% fetal bovine serum (FBS), 2 mM L-glutamine, 100 units/mL penicillin, 100 µg/mL streptomycin, and 0.25 µg/mL amphotericin.

Specific pathogen-free ECEs at 9 days of age were ordered from USDA NVSL and incubated at 37°C with passive humidity.

Primary CEF cells and primary chicken embryo lung (CEL) cells were prepared from embryonated chicken eggs following similar protocols with some specific modifications at certain steps. For primary CEF cells, embryonated chicken eggs at 9–10 days of age were used, while primary CEL cells were derived from 19-day-old chicken embryos. The 9-day-old embryonated chicken eggs ordered from NVSL were incubated in an egg incubator until they reached 19 days old before primary CEL cells were prepared. For the preparation of primary CEF cells, the heads, wings, legs, and internal organs of the prechilled embryos were removed, and the remaining tissues were collected. For the preparation of primary CEL cells, the lung tissues from prechilled embryos were collected. In both cases, the collected tissues were washed three times with PBS (pH 7.4, Thermo Fisher Scientific), cut into 1 cm pieces, washed three times with PBS, and treated with a 0.25% trypsin solution at 37°C for 10–15 min (tissues for CEF) or 25–30 min (tissues for CEL) until the tissue pieces became fluffy. After digestion, the trypsin was removed, and the tissue cells were washed twice with PBS and once with the cell culture media (DMEM supplemented with 10% FBS, 2 mM L-glutamine, 100 units/mL penicillin, 100 µg/mL streptomycin, and 0.25 µg/mL amphotericin). The cells were then resuspended in cell culture media and filtered through a funnel containing eight layers of gauze three times. Subsequently, the cell suspensions were collected, and cells from multiple embryos could be pooled. The prepared cell suspensions were either frozen in liquid nitrogen for future use or freshly aliquoted into 24-well culture plates (1 mL per well) and cultured at 37°C with 5% CO_2_. Typically, a monolayer with over 90% confluence was achieved within 24 hours for primary CEF cells. A single chicken embryo could yield primary fibroblast cells to populate up to 150 wells of 24-well plates. Typically, a monolayer with over 90% confluence was achieved in 4–5 days for primary CEL cells. A single chicken embryo could yield primary lung cells to populate up to 48 wells of 24-well plates.

### Primary isolation of aMPV from clinical samples

Isolation of aMPV-A and aMPV-B from PCR-positive clinical samples was attempted in the Vero cell line, UMNSAH/DF-1 cell line, 9-day-old ECEs, primary CEF cells, and/or primary CEL cells. The clinical samples were first filtered through 0.22 µm surfactant-free cellulose acetate syringe filters (Corning, NY, USA).

The cell lines or primary cells cultured in 24-well plates were washed once with PBS and once with the washing media (DMEM supplemented with 2 mM L-glutamine, 100 units/mL penicillin, 100 µg/mL streptomycin, and 0.25 µg/mL amphotericin). Then, the filtered samples were inoculated into cells (0.1–0.2 mL per well) and incubated at 37°C with 5% CO_2_ for 1.5 hours. The inoculum was removed, and the virus isolation media (DMEM supplemented with 2% FBS, 2 mM L-glutamine, 100 units/mL penicillin, 100 µg/mL streptomycin, and 0.25 µg/mL amphotericin) was added (1 mL per well). The plates were incubated at 37°C with 5% CO_2_. The development of cytopathic effects (CPEs) was checked daily. When ~70%–80% of viral CPEs were observed, the plates were subject to two freeze (−80°C) and thaw (room temperature) cycles before harvesting. If no CPE was observed after 4–5 days, the plates were subject to two freeze-thaw cycles before harvesting. The cell lysates were centrifuged at 2,000 rpm for 10 min and the supernatants were harvested. The supernatants were tested by aMPV-A, -B, and -C real-time RT-PCR as described above. Virus isolation was performed through three passages (P0–P2) in Vero cells or UMNSAH/DF-1 cells and five passages (P0–P4) in primary CEF and CEL cells. In Vero and UMNSAH/DF-1 cells, samples were deemed VI-negative if no CPE was observed after three passages and the third-passage cell culture supernatant tested PCR-negative. In primary CEF and CEL cells, samples were considered VI-negative if no CPE was observed after five passages and the fifth-passage cell culture supernatant was PCR-negative. In contrast, if CPE was observed during the course of five passages and the fifth-passage cell culture supernatant tested PCR-positive for aMPV with Ct ≤20, the samples were considered VI-positive in primary CEF and/or CEL cells. The obtained virus isolates were stored at −80°C until use.

For VI in embryonated chicken eggs, 0.2 mL of each sample was inoculated into the allantoic cavity of three 9-day-old ECE (35), followed by incubation at 37°C with passive humidity for 3–4 days. After chilling the eggs at 4°C for approximately 2 hours, the allantoic fluids were harvested and centrifuged at 2,000 rpm for 10 min. The supernatants were tested by aMPV-A, -B, and -C real-time RT-PCR as described above. For each sample, three passages of VI were conducted. The samples were considered VI-negative if allantoic egg fluids from the third passage tested negative by aMPV RT-PCR.

### Serial passages of aMPV-A and aMPV-B isolates in cell culture

Two aMPV-A isolates (USA/IA55601-6/2024 and USA/IA56509-5/2024) were serially passaged in primary CEL cells, and two aMPV-B isolates (USA/NC20487-GA/2024 and USA/NC23734-GA/2024) were serially passaged in primary CEF cells for 10 passages (P0–P9). The cell culture supernatants at each passage were tested by aMPV-A, -B, and -C real-time RT-PCR.

Two aMPV-A isolates (USA/IA55601-6/2024 and USA/IA56509-5/2024) at P4 obtained from primary CEL cells were inoculated into Vero cells and serially propagated for 10 passages (P1–P10) with the first passage in Vero cells designated as P1. Similarly, aMPV-B isolate USA/NC20487-GA/2024 at P5 and USA/NC23734-GA/2024 isolate at P6 obtained from primary CEF cells were inoculated into Vero cells and serially propagated for 10 passages (P1–P10) with the first passage in Vero cells designated as P1. The cell culture supernatants at each passage were tested by aMPV-A, -B, and -C real-time RT-PCR and titrated in Vero cells. Immunofluorescence staining was also performed on aMPV-inoculated Vero cells to verify virus replication.

### Titration of aMPV-A and aMPV-B isolates in Vero cells

The infectious titers of aMPV-A and aMPV-B isolates adapted in Vero cells (P1–P10) were determined in Vero cells. Each virus isolate at each passage was 10-fold serially diluted, and 100 µL of each dilution was inoculated into Vero cells grown in 96-well plates with triplicate wells per dilution. CPE was recorded for each well daily up to 4–5 days post-inoculation. Immunofluorescence staining described in the section Immunofluorescence staining below was conducted to verify virus replication. Virus titers were calculated according to the Reed and Muench method (36) and expressed as TCID_50_/mL.

### Immunofluorescence staining

Mock-infected and aMPV-infected Vero cells cultured in 96-well plates were fixed with 80% cold acetone. After air drying, the plates were rinsed with 100 µL of PBS and incubated with 100 µL of 40-fold diluted turkey serum samples for 1 hour at 37°C. Three types of turkey serum samples were used: (i) aMPV enzyme-linked immunosorbent assay (ELISA) antibody-negative turkey serum, (ii) aMPV-A ELISA antibody-positive serum from a turkey that was PCR-positive only for aMPV-A, and (iii) aMPV-B ELISA antibody-positive serum from a turkey that was PCR-positive only for aMPV-B. The avian metapneumovirus ELISA test (IDEXX, Westbrook, ME, USA) was performed according to the manufacturer’s protocol.

After incubation, the plates were washed three times with PBS (200 µL/well). Then, 50 µL of 400-fold diluted goat anti-turkey IgG conjugated to fluorescein isothiocyanate (Thermo Fisher Scientific) was added to each well. Following a 45 min incubation at 37°C, the plates were washed three more times with PBS (200 µL/well). The plates were read under a fluorescence microscope.

### Whole-genome sequencing and sequence analysis

The whole-genome sequences of selected aMPV-A and aMPV-B isolates were determined via next-generation sequencing (NGS). Briefly, nucleic acids were extracted from the aMPV-A isolate USA/IA55601-6/2024 at P1 and P9 in primary CEL cells and at P4 and P10 in Vero cells, the aMPV-A isolate USA/IA56509-5/2024 at P3 and P9 in primary CEL cells and at P4 and P10 in Vero cells, the aMPV-B isolate USA/NC20487-GA/2024 at P3 and P9 in primary CEF cells and at P4 and P10 in Vero cells, and the aMPV-B isolate USA/NC23734-GA/2024 at P1 and P9 in primary CEF cells and at P4 and P10 in Vero cells, using MagMAX Pathogen RNA/DNA Kit (Thermo Fisher Scientific) on a Kingfisher Flex instrument (Thermo Fisher Scientific) following the manufacturer’s instructions. cDNA was synthesized using NEXTflex Rapid RNA-Seq Kit (Bioo Scientific Corp, Austin, TX, USA). Sequencing library was prepared using Nextera XT DNA library preparation kit (Illumina, San Diego, CA, USA). The libraries were sequenced on the Illumina MiSeq platform available at the ISU VDL with the 500-Cycle v.2 Reagent Kit (Illumina). Bioinformatics analysis was performed following the previously described procedures for other RNA viruses (37–39).

In addition to the aMPV-A and aMPV-B sequences determined in this study, 46 whole-genome sequences of aMPV retrieved from GenBank on 8 January 2025 were included for analysis. These 46 sequences included 13 aMPV-A, 18 aMPV-B, 14 aMPV-C, and 1 aMPV-D. Nucleotide identities were calculated by the MAFFT alignment of the MegAlign Pro 17 program in the DNASTAR Lasergene 17 software. For phylogenetic analysis, sequences were first aligned using the progressive method (FFT-NS-2) in MAFFT v.7.407 (40), and then the phylogenetic trees based on aMPV-G gene nucleotides and complete genome sequences were inferred from the alignment results using maximum likelihood with a stochastic algorithm in IQ-TREE v.2.2.2.6 with 1,000 bootstrap replicates (41).

## RESULTS

### Summary of aMPV RT-PCR results at ISU VDL from January through November 2024

The 2,204 clinical samples tested by aMPV-A, -B, and -C real-time RT-PCR at ISU VDL from January to November 2024 included 1,158 turkey samples, 936 chicken samples, and 110 other breed samples. All samples were PCR-negative for aMPV-C. Among turkey samples, 591/1,158 (51.04%) were PCR-positive for aMPV-A and/or aMPV-B (410/1,158 [35.41%] aMPV-A positive only, 165/1,158 [14.25%] aMPV-B positive only, and 16/1,158 [1.38%] positive for both aMPV-A and aMPV-B) (Table 1). In contrast, only 146/936 (15.6%) of chicken samples were PCR-positive for aMPV-A and/or aMPV-B (91/936 [9.72%] aMPV-A positive only, 49/936 [5.24%] aMPV-B positive only, and 6/936 [0.64%] positive for both aMPV-A and aMPV-B) (Table 1). Regarding 110 samples of other breeds, only two samples with unknown breed were aMPV-A PCR-positive (Table 1). The number of samples and their PCR-positive rates were further analyzed at different age categories and are summarized in Table 1. aMPV-A and aMPV-B were detected across different ages of turkeys and chickens with varying positive rates. For turkeys, more clinical samples in the age of 3–24 weeks were received, and relatively higher positive rates were detected in this age range compared to other age categories. For chickens, most of the received samples were in the age of ≥24 weeks and no clear trends of positive rates were observed across different age categories.

**TABLE 1:** Summary of aMPV real-time RT-PCR results at ISU VDL from January to November 2024^*a*^^,^^*b*^

Breed	aMPV RT-PCR result	Number of samples (% at each age category)					
		All age	<3 weeks	3 to <8 weeks	8 to <24 weeks	≥24 weeks	Unknown age
Turkey	aMPV-A Pos only	410 (35.41%)	38 (22.49%)	112 (36.60%)	208 (39.62%)	0	52 (41.94%)
	aMPV-B Pos only	165 (14.25%)	7 (4.14%)	65 (21.24%)	75 (14.29%)	11 (32.35%)	7 (5.64%)
	aMPV-A and -B Pos	16 (1.38%)	0	11 (3.60%)	5 (0.95%)	0	0
	aMPV-A and -B Neg	567 (48.96%)	124 (73.37%)	118 (38.56%)	237 (45.14%)	23 (67.65%)	65 (52.42%)
	**Subtotal**	**1,158** (**100%**)	**169** (**100%**)	**306** (**100%**)	**525** (**100%**)	**34** (**100%**)	**124** (**100%**)
Chicken	aMPV-A Pos only	91 (9.72%)	2 (16.67%)	7 (20.00%)	9 (12.33%)	73 (9.45%)	0
	aMPV-B Pos only	49 (5.24%)	0	2 (5.71%)	6 (8.22%)	27 (3.49%)	14 (32.56%)
	aMPV-A and -B Pos	6 (0.64%)	0	1 (2.86%)	4 (5.48%)	1 (0.13%)	0
	aMPV-A and -B Neg	790 (84.40%)	10 (83.33%)	25 (71.43%)	54 (73.97%)	672 (86.93%)	29 (67.44%)
	**Subtotal**	**936** (**100%**)	**12** (**100%**)	**35** (**100%**)	**73** (**100%**)	**773** (**100%**)	**43** (**100%**)
Other breed	aMPV-A Pos only	2 (1.82%)	0	0	0	0	2 (4.76%)
	aMPV-B Pos only	0	0	0	0	0	0
	aMPV-A and -B Pos	0	0	0	0	0	0
	aMPV-A and -B Neg	108 (98.18%)	0	5 (100%)	0	63 (100%)	40 (95.24%)
	**Subtotal**	**110** (**100%**)	**0**	**5** (**100%**)	**0**	**63** (**100%**)	**42** (**100%**)
**All**	**Total**	**2,204**	**181**	**346**	**598**	**870**	**209**

^*a*^ Notes: All samples tested negative for aMPV-C by real-time RT-PCR. Other breed includes pheasant (*n* = 3), wild bird (*n* = 100), and unknown (*n* = 7) according to the information provided on the submission forms.

^*b*^ Bold value indicates subtotal or total numbers.

The state distributions of tested clinical samples are summarized in Table 2. Among the turkey samples submitted to the ISU VDL for aMPV testing, the top 3 states of origin were Iowa, Ohio, and North Carolina. In Iowa, 228/466 (48.93%) and 22/466 (4.72%) of turkey samples were aMPV-A and aMPV-B PCR-positive, respectively, clearly indicating that aMPV-A was more frequently detected than aMPV-B in Iowa turkeys. In Ohio, similar rates of turkey samples were PCR-positive for aMPV-A (78/243 [32.10%]) and aMPV-B (61/243 [25.10%]). In North Carolina, more turkey samples were positive for aMPV-B (50/164 [30.49%]) than for aMPV-A (1/164 [0.61%]). Among the chicken samples submitted to the ISU VDL for aMPV testing, the top states of origin were North Carolina, Indiana, and Arkansas, followed by Missouri, Oklahoma, Iowa, Maryland, Tennessee, and Ohio. More chicken samples were positive for aMPV-A than aMPV-B in Arkansas, Missouri, and Oklahoma, similar number of chicken samples were positive for aMPV-A and aMPV-B in Indiana and Ohio, and more chicken samples were positive for aMPV-B than aMPV-A in North Carolina.

**TABLE 2:** State distributions of clinical samples tested for aMPV by RT-PCR at ISU VDL from January to November 2024^*a*^^,*b*^

State	Number of turkey samples			Number of chicken samples			Number of other breed samples			Total
	Subtotal	aMPV-A PCR-Pos (%)	aMPV-B PCR-Pos (%)	Subtotal	aMPV-A PCR-Pos (%)	aMPV-B PCR-Pos (%)	Subtotal	aMPV-A PCR-Pos (%)	aMPV-B PCR-Pos (%)	
Arkansas	0	0	0	123	31 (25.20%)	1 (0.81%)	0	0	0	123
California	17	9 (52.94%)	0	13	7 (53.85%)	1 (7.69%)	0	0	0	30
Colorado	0	0	0	2	0	0	0	0	0	2
Georgia	0	0	0	12	0	6 (50.00%)	0	0	0	12
Iowa	466	228 (48.93%)	22 (4.72%)	69	1 (1.45%)	6 (8.70%)	101	0	0	636
Illinois	3	0	0	5	1	0	0	0	0	8
Indiana	48	23 (47.92%)	9 (18.75%)	167	7 (4.19%)	7 (4.19%)	0	0	0	215
Maryland	0	0	0	54	0	0	0	0	0	54
Michigan	15	5 (33.33%)	10 (66.67%)	10	2	3	0	0	0	25
Minnesota	41	6 (14.63%)	15 (36.59%)	4	0	0	2	0	0	47
Missouri	1	0	0	94	14 (14.89%)	1 (1.06%)	0	0	0	95
Montana	0	0	0	0	0	0	1	0	0	1
North Carolina	164	1 (0.61%)	50 (30.49%)	184	0	11 (5.98%)	0	0	0	348
Ohio	243	78 (32.10%)	61 (25.10%)	50	8 (16.00%)	12 (24.00%)	0	0	0	293
Oklahoma	0	0	0	72	25 (34.72%)	0	0	0	0	72
South Carolina	1	0	1	12	0	0	0	0	0	13
South Dakota	2	0	0	0	0	0	0	0	0	2
Tennessee	0	0	0	53	0	5 (9.43%)	0	0	0	53
Texas	0	0	0	1	0	0	0	0	0	1
Virginia	0	0	0	3	0	1	0	0	0	3
Wisconsin	7	5	0	3	0	1	0	0	0	10
West Virginia	11	0	0	0	0	0	0	0	0	11
Unknown	139	71 (51.08%)	13 (9.35%)	5	1	0	6	2	0	150
**Total**	**1158**	**426** (**36.79%**)	**181** (**15.63%**)	**936**	**97** (**10.36%**)	**55** (**5.88%**)	**110**	**2** (**1.82%**)	**0**	**2,204**

^*a*^ Note: The positive rate (%) is shown only when the subtotal exceeds 10 and there is at least one positive case.

^*b*^ Bold value indicates subtotal or total numbers.

For both turkeys and chickens, the two most common specimen types submitted to the ISU VDL for aMPV RT-PCR testing were oropharyngeal swabs (721/1158 [62.26%] for turkeys and 474/936 [50.64%] for chickens) and tracheal swabs (251/1158 [21.68%] for turkeys and 250/936 [26.71%] for chickens) (Table 3). For turkeys, 34 samples were labeled as “tracheal/oropharyngeal swab” on the submission forms and were listed separately in Table 3. Including them, the total number of turkey oropharyngeal and/or tracheal swabs increases to 1,006/1,158 (86.87%). In turkeys, 376/721 (52.15%) of oropharyngeal swabs and 134/251 (53.39%) of tracheal swabs were PCR-positive for aMPV-A and/or aMPV-B. In contrast, in chickens, 69/474 (14.56%) of oropharyngeal swabs and 51/250 (20.40%) of tracheal swabs were PCR-positive for aMPV-A and/or aMPV-B.

**TABLE 3:** Specimen types of clinical samples tested for aMPV by RT-PCR at ISU VDL from January to November 2024^*b*^

Specimen type	Turkey: number of samples by status of aMPV PCR					Chicken: number of samples by status of aMPV PCR				
	aMPV-A Pos only (Ct range)	aMPV-B Pos only (Ct range)	aMPV-A and -B Pos (Ct range)	aMPV-A and -B Neg [%]	Total	aMPV-A Pos only (Ct range)	aMPV-B Pos only (Ct range)	aMPV-A and -B Pos (Ct range)	aMPV-A and -B Neg [%]	Total
Oropharyngeal swab	265 (17.5–36.9)	105 (16.5–36.9)	6 (16.2–36.8)	345 [47.85%]	**721**	41 (18.1–36.6)	27 (17.7–36.9)	1 (27.8–29.3)	405 [85.44%]	**474**
Trachea	8 (20.8–34.2)	5 (18.6–31.4)	0	28 [68.29%]	**41**	1 (31.1)	2 (20.9–29.2)	3 (25.4–36.7)	16 [72.73%]	**22**
Tracheal swab	92 (19.1–36.9)	33 (20.1–36.6)	9 (19.1–36.1)	117 [46.61%]	**251**	38 (18.4–36.5)	12 (20.7–35.5)	1 (22.3–22.5)	199 [79.60%]	**250**
Tracheal/Oropharyngeal swab	18 (21.6–35.2)	2 (31.3–31.7)	0	14 [41.18%]	**34**	0	1 (29.1)	0	3 [75%]	**4**
Turbinates	8 (21.5–30.7)	6 (20.4–36.4)	1 (19.2–21.5)	21 [58.33%]	**36**	0	0	0	1 [100%]	**1**
Lung	5 (28.6–30.4)	0	0	5 [50.00%]	**10**	0	0	0	0	**0**
Serum	0	0	0	0	**0**	0	0	0	150 [100%]	**150**
Other^*a*^	14 (19.5–36.2)	14 (13.1–35.8)	0	37 [56.92%]	**75**	11 (24.2-36.5)	7 (33.4-36.7)	1 (18.5-22.4)	16 [45.71%]	**35**
**Total**	**410**	**165**	**16**	**567 [48.96%]**	**1,158**	**91**	**49**	**6**	**790 [84.40%]**	**936**

^*a*^ Other specimen types for turkey include unspecified swab (n=24), joint swab (n = 5), choanal swab (n = 8), environmental (n = 8), unspecified fluid (n = 6), nasal cavity (n = 1), sinus (n = 3), liver tissue (n = 1), heart tissue (n = 1), semen (n = 2), and unspecified tissue (n = 6). Other specimen types for chicken include unspecified swab (n = 8), unspecified fluid (n = 17), nasal cavity (n = 2), sinus (n = 3), and unspecified tissue (n = 5).

^*b*^ Bold value indicates subtotal or total numbers.

**Fig 1:**
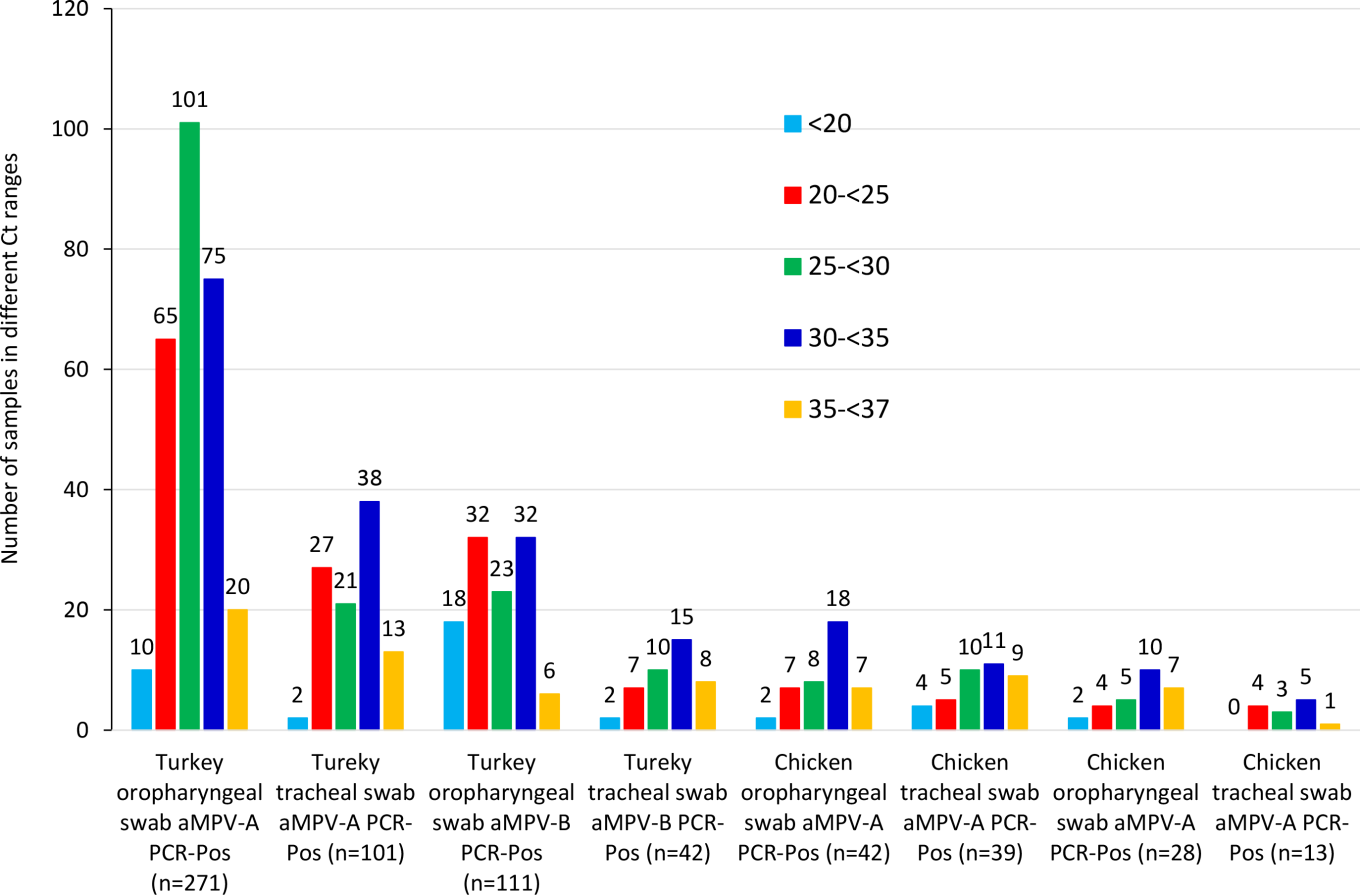
Distributions of aMPV-A and aMPV-B RT-PCR cycle of threshold (Ct) values in oropharyngeal swabs and tracheal swabs. On the *x*-axis, eight different categories are shown. On the *y*-axis, the number of samples in different Ct ranges is shown.

In both turkeys and chickens, samples had varying aMPV RT-PCR Ct ranges regardless of specimen types (Table 3). For the two most common specimen types (oropharyngeal swabs and tracheal swabs), the distributions of aMPV-A and aMPV-B RT-PCR Ct values are summarized in Fig. 1. Among turkey oropharyngeal swabs and tracheal swabs that tested PCR-positive for aMPV-A, the majority had Ct values in the range of 20–35: 241/271 (88.93%) oropharyngeal swabs and 86/101 (85.15%) tracheal swabs; very few samples had Ct value <20: 10/271 (3.69%) oropharyngeal swabs and 2/101 (1.98%) tracheal swabs. In contrast, among the turkey oropharyngeal swabs that tested PCR-positive for aMPV-B, a relatively higher proportion of samples (18/111 [16.22%]) had Ct values <20. The number of samples in other categories presented in Fig. 1 was comparatively small.

### Primary isolation of aMPV-A and aMPV-B from clinical samples

Initially, 11 aMPV-A PCR-positive samples in the Ct range of 18.9–31.1 and 36 aMPV-B PCR-positive samples in the Ct range of 16.5–34.1 were directly subject to VI attempts in the continuous Vero cell line. The cell lysates at three passages (P0–P2) were tested by aMPV-A and aMPV-B real-time RT-PCR. Due to the absence of CPE and the negative PCR results on the third-passage cell lysates, VI was considered negative for these samples in the Vero cell line.

Subsequently, 12 aMPV-A PCR-positive samples in the Ct range of 18.9–24.8 and 12 aMPV-B PCR-positive samples in the Ct range of 16.5–28 were directly inoculated into the continuous UMNSAH/DF-1 cell line for VI attempts. No CPE was observed during three passages (P0–P2), and the third-passage cell lysates tested PCR-negative for aMPV. Therefore, VI was considered unsuccessful for these samples in the UMNSAH/DF-1 cell line.

Three aMPV-A PCR-positive samples in the Ct range of 18.9–25.1 and two aMPV-B PCR-positive samples in the Ct range of 20.3–22.3 were also directly inoculated into 9-day-old ECEs via allantoic cavity route for VI attempts. After three passages (P0–P2), the harvested allantoic fluids were negative by aMPV-A and aMPV-B RT-PCR, and VI was considered negative.

**Fig 2:**
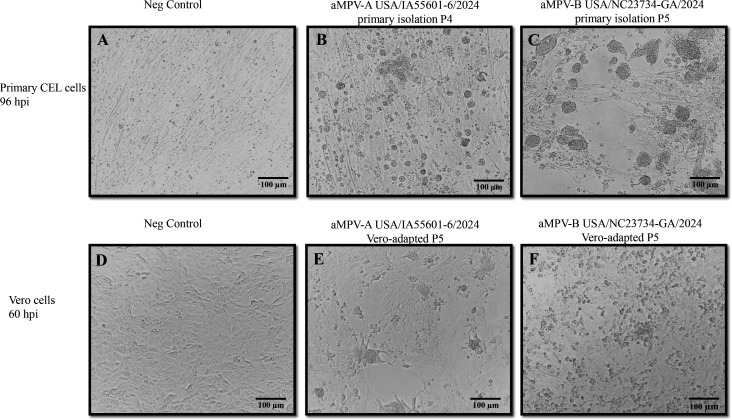
CPE images. Mock-infected primary CEL cells (**A**), primary CEL cells infected with the aMPV-A USA/IA55601-6/2024 primary isolate P4 (**B**), and primary CEL cells infected with the aMPV-B USA/NC23734-GA/2024 primary isolate P5 (**C**) at 96 hours post-infection (hpi) are shown. Mock-infected Vero cells (**D**), Vero cells infected with the Vero cell-adapted aMPV-A isolate P5 (**E**), and Vero cells infected with the Vero cell-adapted aMPV-B isolate P5 (**F**) at 60 hpi are shown.

Next, virus isolation was attempted in primary CEF cells and/or primary CEL cells prepared in our laboratory. Forty-nine aMPV-A PCR-positive clinical samples in the Ct range of 17.5–32.0 (47 samples positive for aMPV-A only and two samples positive for both aMPV-A and aMPV-B) were subject to VI attempts in primary cells. As shown in Table S1, four samples (USA/IA55601-6/2024, USA/IA56509-5/2024, USA/OH44164-1/2024, and USA/IN39902-1/2024) with the Ct values of 17.6, 18.9, 22.5, and 24.8 were aMPV-A VI-positive in either primary CEF cells or primary CEL cells or both. Similarly, 42 aMPV-B PCR-positive clinical samples in the Ct range of 16.5–33.8 (40 samples positive for aMPV-B only and two samples positive for both aMPV-A and aMPV-B) were subject to VI attempts in primary cells. Three samples (USA/NC23734-GA/2024, USA/NC20487-GA/2024, and USA/NC39727-GB/2024) with the Ct values of 18.6, 19.7, and 21.1 were aMPV-B VI-positive in either primary CEF cells alone or in both primary CEF and primary CEL cells (Table S2). Regarding the two clinical samples that were PCR-positive for both aMPV-A and aMPV-B (USA/OH44164-1/2024 and USA/OH46642-1/2024), the sample USA/OH44164-1/2024 was aMPV-A VI-positive and aMPV-B VI-negative, whereas the sample USA/OH46642-1/2024 was both aMPV-A and aMPV-B VI-negative. For four aMPV-A and three aMPV-B VI-positive samples, CPE was not apparent during P0–P2; however, from P3 onward, CPE, characterized by rounded and floating cells, appeared in primary CEF and CEL cells. Figure 2 shows the representative images of primary CEL cells: mock-infected (Fig. 2A), aMPV-A USA/IA56601-6/2024 primary isolate P4-infected (Fig. 2B), and aMPV-B USA/NC23734-GA/2024 primary isolate P5-infected (Fig. 2C). The specimen types of the seven VI-positive samples are oropharyngeal swab, tracheal swab, or trachea (Tables S1 and S2). Notably, many clinical samples had low Ct values for aMPV-A or aMPV-B but were VI-negative (Tables S1 and S2).

Two aMPV-A isolates (USA/IA55601-6/2024 and USA/IA56509-5/2024) and two aMPV-B isolates (USA/NC20487-GA/2024 and USA/NC23734-GA/2024) were selected for 10 serial passages (P0–P9) in primary CEL cells and primary CEF cells, respectively. As shown in Table S3, two aMPV-A and two aMPV-B isolates grew efficiently upon serial passages in primary CEL and primary CEF cells, respectively, as evidenced by consistent low PCR Ct values during P2–P9.

### Adaptation of aMPV-A and aMPV-B isolates obtained in primary cells to grow in continuous Vero cell line

Two aMPV-A isolates (USA/IA55601-6/2024 and USA/IA56509-5/2024) at P4 obtained from primary CEL cells were serially propagated in Vero cells for 10 passages (P1–P10). The aMPV-B isolates USA/NC20487-GA/2024 at P5 and USA/NC23734-GA/2024 isolate at P6 obtained from primary CEF cells were serially propagated in Vero cells for 10 passages (P1–P10). For the aMPV-A isolate USA/IA55601-6/2024, no CPE was observed at P1, mild CPE appeared at P2, and extensive CPE developed from P3 to P10 in Vero cells. Similarly, for the aMPV-A isolate USA/IA56509-5/2024, no CPE was observed at P1 and P2, mild CPE appeared at P3, and extensive CPE was observed during P5–P10 in Vero cells. In contrast, the two aMPV-B isolates (USA/NC20487-GA/2024 and USA/NC23734-GA/2024) exhibited extensive CPE throughout P1–P10 in Vero cells. As exemplified in Fig. 2E and F, the characteristic CPE observed in Vero cells inoculated with aMPV-A or aMPV-B included cell rounding and enlargement, with prominent syncytial formation in cells infected with the aMPV-A isolate (Fig. 2E). In contrast, no CPE was observed in mock-inoculated Vero cells (Fig. 2D). Based on the CPE development, it appeared that the aMPV-A isolate USA/IA55601-6/2024 propagated faster than the aMPV-A isolate USA/IA56509-5/2024, while both aMPV-B isolates USA/NC20487-GA/2024 and USA/NC23734-GA/2024 grew efficiently in Vero cells.

**Fig 3:**
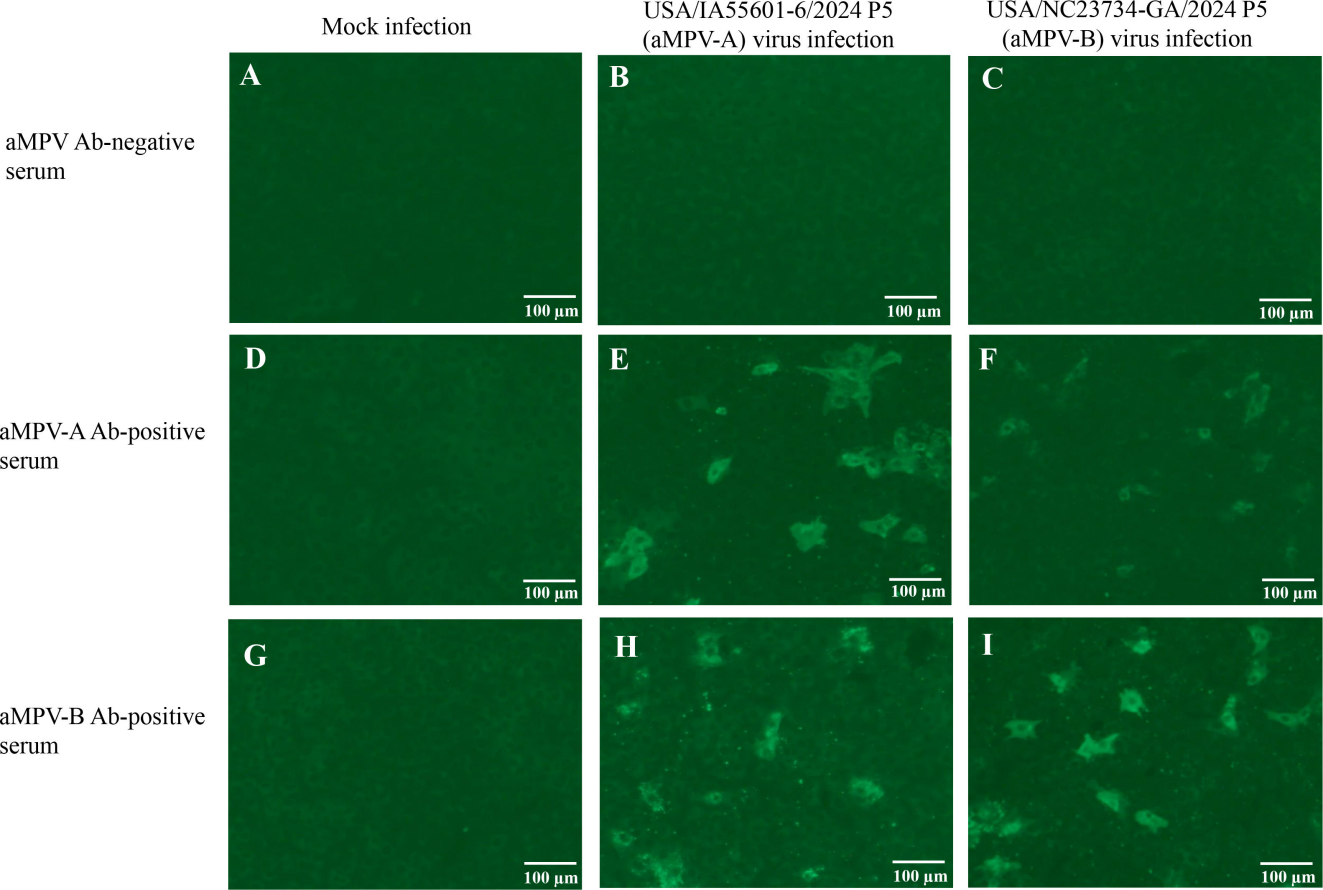
Immunofluorescence staining of mock- or virus-infected cells. Vero cells inoculated with culture medium (mock), aMPV-A isolate USA/IA55601-6/2024, or aMPV-B isolate USA/NC23734-GA/2024 were fixed at 24 hours post-inoculation. The cells were stained with aMPV antibody-negative turkey serum (**A–C**), aMPV-A antibody-positive turkey serum (**D–F**), and aMPV-B antibody-positive turkey serum (**G–I**), followed by staining with goat anti-turkey IgG conjugated to fluorescein isothiocyanate.

Replication of Vero cell-adapted aMPV-A and aMPV-B isolates was confirmed by immunofluorescence staining, as shown in Fig. 3. aMPV antibody-negative turkey serum showed no specific staining in mock-infected, aMPV-A virus-infected, or aMPV-B virus-infected Vero cells (Fig. 3A through C). aMPV-A antibody-positive turkey serum did not stain mock-infected cells (Fig. 3D) but stained both aMPV-A virus-infected (Fig. 3E) and aMPV-B virus-infected (Fig. 3F) cells. Similarly, aMPV-B antibody-positive turkey serum did not stain mock-infected cells (Fig. 3G) but stained both aMPV-A virus-infected (Fig. 3H) and aMPV-B virus-infected (Fig. 3I) cells.

The Vero cell-adapted aMPV-A and aMPV-B isolates at P1–P10 were also tested by aMPV real-time RT-PCR. As shown in Table 4, P1–P10 of the two aMPV-A isolates and two aMPV-B isolates all had low PCR Ct values, indicating that these virus isolates grew efficiently in Vero cells after adaptation. The two aMPV-B isolates overall had relatively lower Ct values compared to the two aMPV-A isolates during P1–P10 in Vero cells. The aMPV-A isolate USA/IA55601-6/2024 had infectious titers of ~10^1^–10^3^ TCID_50_/mL at P1 and P2, and the aMPV-A isolate USA/IA56509-5/2024 had infectious titers of ~10^0^–10^1^ TCID_50_/mL at P1–P3, but both isolates had titers of ~10^4^–10^6^ TCID_50_/mL at P4–P10 (Table 4), demonstrating that the aMPV-A isolates could be adapted to grow efficiently in Vero cells after a few passages. The two aMPV-B isolates (USA/NC20487-GA/2024 and USA/NC23734-GA/2024) had decent titers from P1 and maintained the titers of ~10^4^–10^6^ TCID_50_/mL during P1–P10 (Table 4), suggesting that these two aMPV-B isolates were readily adapted to grow efficiently in Vero cells.

**TABLE 4:** RT-PCR testing and TCID_50_ titers of aMPV-A and aMPV-B isolates serially passaged in Vero cells

Virus isolate	Subtype	Passage	aMPV-A PCR Ct	aMPV-B PCR Ct	aMPV-C PCR Ct	TCID_50_/mL in Vero cells
USA/IA55601-6/2024	A	P1	16.92	≥40	≥40	1.47 × 10^1^
		P2	13.87	≥40	≥40	1.47 × 10^3^
		P3	15.43	≥40	≥40	1.47 × 10^4^
		P4	13.59	≥40	≥40	3.16 × 10^4^
		P5	14.52	≥40	≥40	6.81 × 10^5^
		P6	15.65	≥40	≥40	6.81 × 10^5^
		P7	14.84	≥40	≥40	3.16 × 10^5^
		P8	15.11	≥40	≥40	3.16 × 10^6^
		P9	15.80	≥40	≥40	3.16 × 10^6^
		P10	15.60	≥40	≥40	6.81 × 10^5^
USA/IA56509-5/2024	A	P1	18.42	≥40	≥40	6.81 × 10^0^
		P2	16.72	≥40	≥40	1.47 × 10^1^
		P3	14.87	≥40	≥40	3.16 × 10^1^
		P4	12.66	≥40	≥40	1.47 × 10^4^
		P5	14.10	≥40	≥40	6.81 × 10^4^
		P6	15.53	≥40	≥40	3.16 × 10^5^
		P7	14.32	≥40	≥40	1.47 × 10^5^
		P8	15.69	≥40	≥40	3.16 × 10^6^
		P9	15.50	≥40	≥40	1.47 × 10^6^
		P10	15.07	≥40	≥40	6.81 × 10^5^
USA/NC20487-GA/2024	B	P1	≥40	11.82	≥40	3.16 × 10^5^
		P2	≥40	12.77	≥40	6.81 × 10^4^
		P3	≥40	13.44	≥40	1.47 × 10^5^
		P4	≥40	12.92	≥40	1.47 × 10^6^
		P5	≥40	13.84	≥40	1.47 × 10^5^
		P6	≥40	14.37	≥40	1.47 × 10^6^
		P7	≥40	13.30	≥40	3.16 × 10^5^
		P8	≥40	12.25	≥40	3.16 × 10^6^
		P9	≥40	12.80	≥40	1.47 × 10^6^
		P10	≥40	12.80	≥40	1.47 × 10^6^
USA/NC23734-GA/2024	B	P1	≥40	9.30	≥40	3.16 × 10^5^
		P2	≥40	11.40	≥40	6.81 × 10^4^
		P3	≥40	12.19	≥40	1.47 × 10^4^
		P4	≥40	10.33	≥40	1.47 × 10^6^
		P5	≥40	12.06	≥40	3.16 × 10^5^
		P6	≥40	12.96	≥40	3.16 × 10^5^
		P7	≥40	11.17	≥40	6.81 × 10^5^
		P8	≥40	10.91	≥40	1.47 × 10^6^
		P9	≥40	13.00	≥40	3.16 × 10^6^
		P10	≥40	12.90	≥40	1.47 × 10^6^

### Genetic characterization of aMPV-A and aMPV-B isolates during serial passages in cell culture

In order to determine if the aMPV-A and aMPV-B isolates were genetically stable during serial passages in cell culture, the whole-genome sequences of two aMPV-A isolates and two aMPV-B isolates at different passages were determined via NGS, and the results are summarized in Table 5 and 6. The genomic organization of these viruses was similar to other aMPVs and included the 3´ UTR-N–P–M–F–M2.1–M2.2–SH–G–L-5′ UTR.

**TABLE 5:** Nucleotide and amino acid changes of aMPV-A isolates during serial passages in cell culture^*d*^

Genome region (nucleotide position)^*a*^	Encoded protein	Nucleotide in^*a*^					Amino acid in^*b*^				
		Position	Primary cells P1 or P3^*c*^	Primary cells P9	Vero cells P4	Vero cells P10	Position	Primary cells P1 or P3^*c*^	Primary cells P9	Vero cells P4	Vero cells P10
aMPV-A USA/IA55601-6/2024											
3' UTR (1–41)	Not applicable										
N (42–1,217)	Nucleoprotein	959	A	G	G	A					
P (1,242–2,078)	Phosphoprotein	1,398	C	C	T	C	53	P	P	S	P
M (2,104–2,868)	Matrix protein										
F (2,936–4,552)	Fusion protein	3,903	A	G	G	G	323	E	G	G	G
M2.1 (4,579–5,139)	M2.1 protein										
M2.2 (5,096–5,317)	M2.2 protein										
SH (5,370–5,894)	Small hydrophobic protein										
Intergenic region	Not applicable	5,964	G	G	G	A					
G (5,977–7,152)	Surface glycoprotein										
L (7,238–13,252)	Large polymerase	11,118	A	G	G	G	1,294	K	R	R	R
5' UTR (13,253–13,302)	Not applicable										
aMPV-A USA/IA56509-5/2024											
3' UTR (1–41)	Not applicable										
N (42–1,217)	Nucleoprotein										
P (1,242–2,078)	Phosphoprotein										
M (2,104–2,868)	Matrix protein	2,830	A	A	A	T	243	N	N	N	Y
F (2,936–4,552)	Fusion protein	3,902	G	G	G	A	323	E	E	E	K
M2.1 (4,579–5,139)	M2.1 protein										
M2.2 (5,096–5,317)	M2.2 protein										
SH (5,370–5,894)	Small hydrophobic protein										
Intergenic region	Not applicable	5,939	T	T	A	T					
G (5,977–7,152)	Surface glycoprotein										
L (7,238–13,252)	Large polymerase	8,579	T	T	T	C	448	F	F	F	L
		10,958	C	C	C	T					
5' UTR (13,253–13,302)	Not applicable										

^*a*^ Nucleotides are numbered according to the respectivesequences of aMPV-A USA/IA55601-6/2024 (GenBank PV067037) and aMPV-A USA/IA56509-5/2024 (GenBank PV067041).

^*b*^ Only nonsynonymous mutations are shown. Amino acids of proteins are numbered according to their locations in the respective proteins.

^*c*^ Primary chicken embryo lung (CEL) cells P1 for aMPV-A USA/IA55601-6/2024 and primary CEL cells P3 for aMPV-A USA/IA56509-5/2024.

^*d*^ Empty cells indicates that no nucleotide changes or amino acid changes were observed in those genes and proteins.

**TABLE 6:** Nucleotide and amino acid changes of aMPV-B isolates during serial passages in cell culture^*d*^

Genome region (nucleotide position)^*a*^	Encoded protein	Nucleotide in^*a*^					Amino acid in^*b*^				
		Position	Primary cells P1 or P3^*c*^	Primary cells P9	Vero cells P4	Vero cells P10	Position	Primary cells P1 or P3^*c*^	Primary cells P9	Vero cells P4	Vero cells P10
aMPV-B USA/NC20487-GA/2024											
3' UTR (1–38)	Not applicable										
N (39–1,214)	Nucleoprotein										
P (1,238–2,077)	Phosphoprotein										
M (2,103–2,867)	Matrix protein										
F (2,928–4,544)	Fusion protein	3,262	T	C	C	C	112	V	A	A	A
		4,110	A	T	T	T	395	N	Y	Y	Y
M2.1 (4,557–5,132)	M2.1 protein										
M2.2 (5,089–5,310)	M2.2 protein										
SH (5,349–5,891)	Small hydrophobic protein										
G (5,997–7,241)	Surface glycoprotein										
L (7,342–13,356)	Large polymerase										
5' UTR (13,357–13,472)	Not applicable										
aMPV-B USA/NC23734-GA/2024											
3' UTR (1–38)	Not applicable										
N (39–1,214)	Nucleoprotein										
P (1,240–2,079)	Phosphoprotein										
M (2,105–2,869)	Matrix protein										
F (2,930–4,546)	Fusion protein										
M2.1 (4,559–5,134)	M2.1 protein										
M2.2 (5,091–5,312)	M2.2 protein	5,212	A	A	A	G	41	E	E	E	G
		5,295	T	T	A	T	69	Y	Y	N	Y
SH (5,351–5,893)	Small hydrophobic protein										
G (5,999–7,243)	Surface glycoprotein	7,041	C	C	T	C	348	P	P	L	P
L (7,344–13,358)	Large polymerase										
5' UTR (13,359–13,474)	Not applicable										

^*a*^ Nucleotides are numbered according to the respective sequences of aMPV-B USA/NC20487-GA/2024 (GenBank PV067045) and aMPV-B USA/NC23734-GA/2024 (GenBank PV067049).

^*b*^ Amino acids of proteins are numbered according to their locations in the respective proteins.

^*c*^ Primary chicken embryo fibroblast (CEF) cells P3 for aMPV-B USA/NC20487-GA/2024 and primary CEF cells P1 for aMPV-B USA/NC23734-GA/2024.

^*d*^ Empty cells indicates that no nucleotide changes or amino acid changes were observed in those genes and proteins.

The aMPV-A isolate USA/IA55601-6/2024 had a genome length of 13,302 nucleotides across all passages tested (primary CEL cells P1 and P9, Vero cells P4 and P10). Their sequences showed 99.97%–99.99% nucleotide identity to each other at the whole genome level, with only five nucleotide substitutions at the positions 959, 1,398, 3,903, 5,964, and 11,118. Of these, the nucleotide change at position 959 was synonymous, the nucleotide change at position 5,964 was located in the intergenic region between the SH and G genes, while the other three nucleotide substitutions (positions 1,398, 3,903, and 11,118) resulted in amino acid changes P53S, E323G, and K1294R in the P, F, and L proteins (Table 5). Similarly, the aMPV-A isolate USA/IA5509-5/2024 also maintained a 13,302-nucleotide genome across all evaluated passages (primary CEL cells P3 and P9, Vero cells P4 and P10), with 99.96%–100% nucleotide identity to each other. Five nucleotide substitutions were identified at the positions 2,830, 3,902, 5,939, 8,579, and 10,958. The position 5,939 was located in the intergenic region between the SH and G genes. The nucleotide change at position 10,958 was synonymous, while the other three nucleotide substitutions (positions 2,830, 3,902, and 8,579) caused amino acid changes N243Y, E323K, and F448L in the M, F, and L proteins (Table 5).

The aMPV-B isolate USA/NC20487-GA/2024 had a genome length of 13,472 nucleotides across all evaluated passages (primary CEF cells P3 and P9, Vero cells P4 and P10), with 99.98%–100% nucleotide identity to each other. Among different passages of this virus isolate, only two nucleotide substitutions were identified (positions 3,262 and 4,110), both of them resulting in amino acid changes V112A and N395Y in the F protein (Table 6). The aMPV-B isolate USA/NC23734-GA/2024 had a genome length of 13,474 nucleotides across all passages tested (primary CEF cells P1 and P9, Vero cells P4 and P10), with 99.98%–99.99% nucleotide identity to each other at the whole genome level. Three nucleotide substitutions were identified at positions 5,212, 5,295, and 7,041, with the resultant three amino acid changes E41G, Y69N, and P348L in the M2.2 and G proteins (Table 6). The aMPV-B isolate USA/NC23734-GA/2024 at all of the evaluated passages had an insertion of two nucleotides “AA” in the intergenic region between N and P genes when compared to the aMPV-B isolate USA/NC20487-GA/2024.

### Sequence comparisons with other aMPV strains

The aMPV-A and aMPV-B sequences determined in this study were compared to 46 other aMPV-A, -B, -C and -D sequences retrieved from GenBank (Table S4). The two aMPV-A isolates (USA/IA55601-6/2024 and USA/IA56509-5/2024) had 99.93%–99.99% (whole genome) and 100% (G gene) nucleotide identity to each other, 99.71%–99.92% (whole genome) and 99.66% (G gene) identity to other US aMPV-A sequences (GenBank PP442011 and PP442012), and 97.04%–99.61% (whole genome) and 94.64%–98.98% (G gene) identity to aMPV-A sequences reported from other countries (see Table S4 for the information of these sequences). Similarly, the two aMPV-B isolates (USA/NC20487-GA/2024 and USA/NC23734-GA/2024) had 99.86%–99.89% (whole genome) and 99.57%–99.65% (G gene) nucleotide identity to each other, 99.81%–99.94% (whole genome) and 99.31%–99.86% (G gene) nucleotide identity to other US aMPV-B sequences, and 97.29%–98.62% (whole genome) and 93.11%–95.87% (G gene) nucleotide identity to aMPV-B sequences reported from other countries. In contrast, lower nucleotide identities were observed between different subtypes, for example, approximately 72%–73% identity between aMPV-A and aMPV-B, between aMPV-A and aMPV-D, or between aMPV-B and aMPV-D, and about 63% identity between aMPV-A and aMPV-C or between aMPV-B and aMPV-C, at the whole-genome level, implying that aMPV-C is distantly related to aMPV-A, -B and -D.

**Fig 4:**
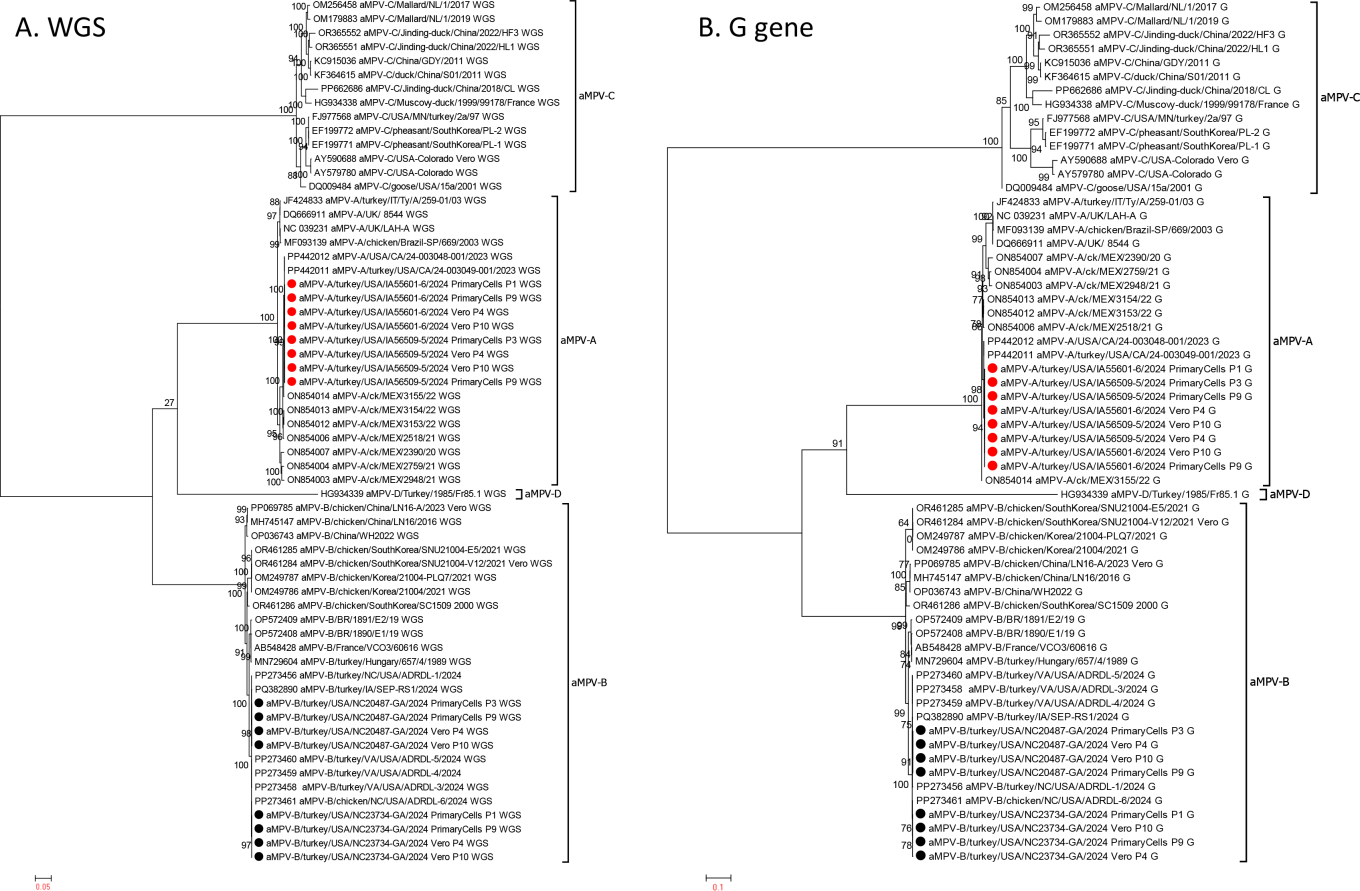
Phylogenetic trees based on the whole-genome sequences (**A**) and the G gene nucleotide sequences (**B**) of aMPV-A and aMPV-B sequences from this study together with 46 aMPV sequences retrieved from GenBank (13 aMPV-A, 18 aMPV-B, 14 aMPV-C, and 1 aMPV-D sequences). The information of these sequences used for phylogenetic analysis is summarized in Table S5. The aMPV-A isolates and the aMPV-B isolates characterized at different passages in cell culture in this study are depicted using solid red circles and solid black circles, respectively.

Phylogenetic analysis clearly demonstrated that the aMPV-A and aMPV-B sequences from this study clustered with other aMPV-A and aMPV-B sequences, respectively, regardless of whether the analysis was based on whole-genome sequences (Fig. 4A) or G gene nucleotide sequences (Fig. 4B). Consistent with the aforementioned nucleotide identity data, phylogenetic trees based on both the whole-genome sequences and the G gene sequences demonstrated that aMPV-C clustered differently from aMPV-A, -B, and -D (Fig. 4A and B).

## DISCUSSION

Since their emergence in the USA for the first time in late 2023 and early 2024, aMPV-A and aMPV-B have continued to be a growing threat to the US poultry industry. However, the detailed epidemiology information was lacking. This study analyzed the detection frequency of aMPV-A and aMPV-B in US poultry based on the RT-PCR data on 2,204 clinical samples (1,158 turkey samples, 936 chicken samples, and 110 other breed samples) submitted to the ISU VDL from January to November 2024. Although the data were sourced from a single diagnostic laboratory, the substantial sample size and representation from ≥22 US states ensure that the findings provide valuable insights into the trends of aMPV-A and aMPV-B detection in US poultry. Among the tested samples, a higher percentage of turkey samples (51.04%) than chicken samples (15.6%) were PCR-positive for aMPV-A and/or aMPV-B (Table 1). Under the conditions of this study, the overall positive rate for aMPV-A was higher than that for aMPV-B across different age groups of turkeys. A similar trend was observed in chickens, although the difference in detection rates between aMPV-A and aMPV-B was less pronounced than in turkeys (Table 1). However, the aMPV-A and aMPV-B positive rates varied by state. For example, in Iowa, the aMPV-A positive rate (228/466 [48.93%]) was considerably higher than the aMPV-B positive rate (22/466 [4.72%]) in turkeys, whereas in North Carolina, the aMPV-B positive rate (50/164 [30.49%]) was markedly higher than the aMPV-A positive rate (1/164 [0.61%]) in turkeys (Table 2). These data provide insight into the distribution of aMPV-A and aMPV-B across different host species and states in the USA. However, the available epidemiological information is insufficient to fully explain why the detection rates varied between species and states.

Among the specimens submitted to the ISU VDL for aMPV RT-PCR testing, oropharyngeal swabs and tracheal swabs were the most common, suggesting these are the preferred specimen types among poultry veterinarians. Notably, PCR-positive oropharyngeal swabs and tracheal swabs exhibited variable Ct values, which may reflect differences in the stage of aMPV infection at the time of sampling. Interestingly, 150 chicken serum samples were submitted for aMPV RT-PCR testing, and none tested positive. Relatively few postmortem tissue samples were received at the ISU VDL for aMPV RT-PCR testing, preventing meaningful assessment of their positive rates and Ct ranges (Table 3).

The ongoing spread of aMPV across major poultry-producing regions in the USA underscores the urgent need for effective vaccines. Although the USDA has approved the importation of several aMPV vaccines manufactured abroad, their protective efficacy against contemporary US aMPV-A and aMPV-B strains has not been reported. To develop inactivated or live attenuated aMPV vaccines based on the US strains through the conventional approaches, it is essential to isolate aMPV-A and aMPV-B that can grow efficiently in cell culture or embryonated chicken/turkey eggs. In previous studies, aMPV VI has been attempted using various approaches. Lu et al. (42) reported successful isolation of aMPV (subtype unreported) from tracheal samples using tracheal organ culture (TOC) prepared from 19- to 20-day-old chick embryos, and the third TOC harvest was successfully adapted to grow in Vero cells. In another study conducted by Goyal et al. (43), aMPV-C VI was attempted in CEF cells, Vero cells, and Quail tumor 35 (QT-35) cells. Four isolates were obtained by inoculation of CEF cells, and one isolate was obtained in QT-35 cells after three to seven blind passages in cell cultures. Vero cells did not yield any isolate on primary isolation from clinical samples. However, all five isolates could be adapted to grow in Vero cells following primary isolation in CEF or QT-35 cells (43). Kwon et al. (9) reported that, when 25 clinical samples (12 aMPV-A PCR-positive and 13 aMPV-B PCR-positive) were directly inoculated into Vero cells followed by serial passages, two aMPV-A isolates were obtained. In Mexico, aMPV-A was successfully isolated from clinical samples using primary chicken embryo lung and trachea mixed culture with subsequent adaptation to Vero cells (44). In China, successful isolation of aMPV-C was achieved by inoculating clinical samples into 11-day-old duck embryos (22) or 8-day-old duck embryos (45) via the yolk sac, followed by further passages in Vero cells.

In this study, we initially attempted aMPV-A and aMPV-B isolation from clinical samples using the Vero cell line, UMNSAH/DF-1 cell line, and embryonated chicken eggs; however, these attempts were unsuccessful. It is important to note that the World Organization for Animal Health recommends isolating aMPV using 6- to 8-day-old embryonated chicken or turkey eggs via the yolk sac inoculation route (46). However, this study utilized 9-day-old embryonated chicken eggs and the allantoic cavity inoculation route, and only a limited number of clinical samples were tested. Therefore, the negative VI results in eggs in this study should be interpreted with caution, and further efforts to isolate aMPV using embryonated eggs are warranted.

Subsequently, we prepared primary CEF and CEL cells from chicken embryos. Using these primary cells, we successfully isolated aMPV-A from four clinical samples and aMPV-B from three clinical samples. Additionally, two aMPV-A isolates (USA/IA55601-6/2024 and USA/IA56509-5/2024) and two aMPV-B isolates (USA/NC20487-GA/2024 and USA/NC23734-GA/2024) exhibited efficient growth during serial passages in primary CEL and primary CEF cells, respectively (Table S3), confirming that these aMPV isolates can be sustainably propagated in primary cells. Nonetheless, the VI success rates were overall low for aMPV-A (4/49 [8.16%] on clinical samples with Ct values of 17.5–32.0) and aMPV-B (3/42 [7.14%] on clinical samples with Ct values of 16.5–33.8) even using primary CEF and/or CEL cells in this study (Tables S1 and S2). The reasons for the low aMPV VI success rates remain unclear. It is likely that multiple factors, such as the amount of infectious virus present in the clinical samples and the virus isolation protocols used, contributed to the outcome. Further research is needed to improve the success rate of aMPV VI.

Due to the labor-intensive process required to prepare primary CEF and CEL cells, it is inconvenient to propagate and titrate aMPV or serially passage the virus in primary cells toward virus attenuation for vaccine development. Therefore, we further adapted two aMPV-A and two aMPV-B isolates obtained in the primary cells to grow in the Vero cell line. The two aMPV-A isolates reached titers of ~10^4^–10^6^ TCID_50_/mL in Vero cells between P4 and P10, while the two aMPV-B isolates achieved similar titers during P1–P10 in Vero cells (Table 4).

Whole-genome sequencing of the two aMPV-A and two aMPV-B isolates indicated that the viruses remained largely genetically stable during 10 serial passages in either primary cells or Vero cells, although they progressively accumulated several nucleotide changes, some of which resulted in amino acid substitutions in various viral proteins. The precise contributions of these nucleotide and amino acid changes to the phenotypic characteristics of aMPV remain to be determined using a reverse genetics approach. It would be valuable to determine the whole-genome sequences of aMPV directly from the original clinical samples to better understand which nucleotide and amino acid changes may arise during the earliest passages in primary cells. Unfortunately, due to multiple initial attempts at virus isolation in different culture systems, the clinical samples from which the aMPV-A isolates (USA/IA55601-6/2024 and USA/IA56509-5/2024) and aMPV-B isolates (USA/NC20487-GA/2024 and USA/NC23734-GA/2024) were derived were no longer available for whole-genome sequencing.

Based on the available data, the US aMPV-A sequences were highly similar to each other, showing 99.71%–99.99% nucleotide identity for the whole genome and 99.66%–100% for the G gene. In comparison, they shared 97.04%–99.61% (whole genome) and 94.64%–98.98% (G gene) identity with 11 aMPV-A sequences from other countries (7, 2, 1, and 1 sequences from Mexico, the UK, Italy, and Brazil, respectively). Among the non-US aMPV-A sequences, some Mexican sequences from 2022 showed relatively high similarity to the US strains, suggesting the possible transborder spillover between the two countries (10, 47). Likewise, the US aMPV-B sequences exhibited 99.81%–99.94% (whole genome) and 99.31%–99.86% (G gene) nucleotide identity among themselves, and 97.29%–98.62% (whole genome) and 93.11%–5.87% (G gene) nucleotide identity compared to 12 aMPV-B sequences from other countries (5, 3, 2, 1, and 1 sequences from South Korea, China, Brazil, France, and Hungary, respectively). Nevertheless, more aMPV-A and aMPV-B sequences from both the US and other countries are needed to enable more comprehensive evolutionary analyses.

The availability of the Vero cell-adapted US aMPV-A and aMPV-B isolates provides a valuable tool for studying viral pathogenesis, determining the infectious doses in turkeys and chickens at different ages, evaluating the effectiveness of disinfectants and antivirals, and developing vaccines. Currently, no MLV vaccines based on aMPV-A or aMPV-B strains of the US origin are commercially available. To address this gap, we have successfully serially passaged aMPV-A and aMPV-B isolates in Vero cells for over 40 passages. These efforts are ongoing, with the goal of developing live attenuated aMPV-A and aMPV-B vaccines to help mitigate the negative impacts of aMPV infections.

In summary, we analyzed the detection frequency of aMPV-A and aMPV-B in US poultry based on the RT-PCR data from this study. Additionally, multiple aMPV-A and aMPV-B isolates associated with the recent outbreaks in the USA were obtained and characterized. The US aMPV-A strains were found to be genetically closely related, as were the US aMPV-B isolates. These US aMPV-A and aMPV-B cell culture isolates provide valuable tools for further characterization of aMPV properties and for enhancing disease control through the development of specific vaccines.

## Data Availability

The whole genome sequences of two aMPV-A and two aMPV-B isolates at different cell culture passages have been deposited to GenBank: aMPV-A USA/IA55601-6/2024 primary cells P1 (PV067035), primary cells P9 (PV067036), Vero cells P4 (PV067037), Vero cells P10 (PV067038); aMPV-A USA/IA56509-5/2024 primary cells P3 (PV067039), primary cells P9 (PV067040), Vero cells P4 (PV067041), Vero cells P10 (PV067042); aMPV-B USA/NC20487-GA/2024 primary cells P3 (PV067043), primary cells P9 (PV067044), Vero cells P4 (PV067045), Vero cells P10 (PV067046); and aMPV-B USA/NC23734-GA/2024 primary cells P1 (PV067047), primary cells P9 (PV067048), Vero cells P4 (PV067049), Vero cells P10 (PV067050).

## SUPPLEMENTAL MATERIAL

Supplemental tables jcm.00333-25-s0001.docx Tables S1 to S4.

ASM does not own the copyrights to Supplemental Material that may be linked to, or accessed through, an article. The authors have granted ASM a non-exclusive, world-wide license to publish the Supplemental Material files. Please contact the corresponding author directly for reuse.
