# Supplementary material for: Isolation and characterization of avian metapneumovirus subtypes A and B associated with the 2024 disease outbreaks among poultry in the USA
Source: J Clin Microbiol. 2025 Jul 10;63(8):e00333-25. doi: 10.1128/jcm.00333-25 (PMC12345154; doi:10.1128/jcm.00333-25)
Supplement: Supplemental tables — Tables S1 to S4. [file jcm.00333-25-s0001.docx]

Supplemental Table S1. Virus isolation (VI) attempts of aMPV-A in primary chicken embryo fibroblast (CEF) and primary chicken embryo lung (CEL) cells

|  | Sample ID | Breed | Sample | PCR Ct on clinical sample /subtype | VI result in primary CEF cells | VI result in primary CEL cells |
| --- | --- | --- | --- | --- | --- | --- |
| 1 | USA/IA55601-4/2024 | Turkey | Oropharyngeal swab | 17.5/A | - | - |
| 2 | USA/IA55601-5/2024 | Turkey | Oropharyngeal swab | 17.6/A | - | - |
| 3 | USA/IA55601-6/2024 | Turkey | Oropharyngeal swab | 17.6/A | - | + |
| 4 | USA/CA13309-53/2024 | Turkey | Nasal turbinates | 18.9/A | - | - |
| 5 | USA/IA55601-3/2024 | Turkey | Oropharyngeal swab | 18.9/A | - | ND* |
| 6 | USA/IA56509-5/2024 | Turkey | Oropharyngeal swab | 18.9/A | - | + |
| 7 | USA/IA56509-1/2024 | Turkey | Oropharyngeal swab | 19.4/A | - | - |
| 8 | USA/CA13309-55/2024 | Turkey | Nasal turbinates | 20.1/A | - | - |
| 9 | USA/IA56509-2/2024 | Turkey | Oropharyngeal swab | 20.1/A | - | ND |
| 10 | USA/CA26913/2024 | Turkey | Sinus tissue | 20.4/A | - | - |
| 11 | USA/42644-2/2024 | Turkey | Oropharyngeal swab | 21.2/A | - | - |
| 12 | USA/37711-1/2024 | Turkey | Oropharyngeal swab | 21.6/A | - | - |
| 13 | USA/42659-2/2024 | Turkey | Oropharyngeal swab | 22/A | - | - |
| 14 | USA/IA56509-3/2024 | Turkey | Oropharyngeal swab | 22.0/A | - | ND |
| 15 | USA/IA66621-6/2024 | Turkey | Oropharyngeal swab | 22.0/A | - | - |
| 16 | USA/IA55601-8/2024 | Turkey | Oropharyngeal swab | 22.4/A | - | ND |
| 17 | USA/IA56509-4/2024 | Turkey | Oropharyngeal swab | 22.4/A | - | ND |
| 18 | USA/OH44164-1/2024 | Chicken | Tracheal swab | 22.5/A; 22.3/B | + (aMPV-A) | - |
| 19 | USA/IA66621-5/2024 | Turkey | Oropharyngeal swab | 22.5/A | - | - |
| 20 | USA/37711-2/2024 | Turkey | Oropharyngeal swab | 22.6/A | - | - |
| 21 | USA42655-2/2024 | Turkey | Oropharyngeal swab | 22.7/A | - | - |
| 22 | USA/IA66621-2/2024 | Turkey | Oropharyngeal swab | 22.7/A | - | - |
| 23 | USA/42659-1/2024 | Turkey | Oropharyngeal swab | 23/A | - | ND |
| 24 | USA/IA66621-4/2024 | Turkey | Oropharyngeal swab | 23.0/A | - | - |
| 25 | USA/37706-1/2024 | Turkey | Oropharyngeal swab | 23.1/A | - | - |
| 26 | USA/IA66621-1/2024 | Turkey | Oropharyngeal swab | 23.2/A | - | - |
| 27 | USA/IA55601-7/2024 | Turkey | Oropharyngeal swab | 23.4/A | - | ND |
| 28 | USA/42644-1/2024 | Turkey | Oropharyngeal swab | 23.8/A | - | - |
| 29 | USA/OH46642-1/2024 | Turkey | Tracheal swab | 23.9/A; 25.4/B | - | - |
| 30 | USA/42643-4/2024 | Turkey | Oropharyngeal swab | 24.1/A | - | - |
| 31 | USA/42647-1/2024 | Turkey | Oropharyngeal swab | 24.3/A | - | - |
| 32 | USA/IN39902-1/2024 | Chicken | Oropharyngeal swab | 24.8/A | + | + |
| 33 | USA/37707-2/2024 | Turkey | Oropharyngeal swab | 24.9/A | - | - |
| 34 | USA/CA16068-GA/2024 | Turkey | Nasal turbinates | 25.1/A | - | - |
| 35 | USA/42645-1/2024 | Turkey | Oropharyngeal swab | 25.2/A | - | - |
| 36 | USA/42651-1/2024 | Turkey | Oropharyngeal swab | 25.3/A | - | - |
| 37 | USA/42655-1/2024 | Turkey | Oropharyngeal swab | 25.3/A | - | - |
| 38 | USA/37709-1/2024 | Turkey | Oropharyngeal swab | 25.4/A | - | - |
| 39 | USA/37710/2024 | Turkey | Oropharyngeal swab | 25.4/A | - | - |
| 40 | USA/42643-2/2024 | Turkey | Oropharyngeal swab | 25.5/A | - | - |
| 41 | USA/42654-2/2024 | Turkey | Oropharyngeal swab | 25.5/A | - | - |
| 42 | USA/42650-1/2024 | Turkey | Oropharyngeal swab | 25.6/A | - | - |
| 43 | USA/42643-1/2024 | Turkey | Oropharyngeal swab | 26/A | - | - |
| 44 | USA/37708-1/2024 | Turkey | Oropharyngeal swab | 26.2/A | - | - |
| 45 | USA/42645-3/2024 | Turkey | Oropharyngeal swab | 26.6/A | - | - |
| 46 | USA/37708-2/2024 | Turkey | Oropharyngeal swab | 26.9/A | - | - |
| 47 | USA/37709-2/2024 | Turkey | Oropharyngeal swab | 27.4/A | - | - |
| 48 | USA/29099-GA/2024 | Turkey | Turbinates | 29.1/A | - | ND |
| 49 | USA/OH36408/2024 | Turkey | Oropharyngeal swab | 32.0/A | - | - |

*ND: Not Done. The samples that were aMPV-A VI-Positive are highlighted in grey color.

Supplemental Table S2. Virus isolation (VI) attempts of aMPV-B in primary chicken embryo fibroblast (CEF) and primary chicken embryo lung (CEL) cells

|  | Cases ID | Breed | Sample | PCR Ct on clinical sample /subtype | VI result in primary CEF cells | VI result in primary CEL cells |
| --- | --- | --- | --- | --- | --- | --- |
| 1 | USA/IA21041-GA/2024 | Turkey | Oropharyngeal swab | 16.5/B | - | - |
| 2 | USA/IA61472-GB/2024 | Chicken | Oropharyngeal swab | 17.7/B | - | - |
| 3 | USA/IA67228-GA/2024 | Turkey | Oropharyngeal swab | 17.7/B | - | - |
| 4 | USA/IA22319-GA/2024 | Turkey | Oropharyngeal swab | 17.9/B | - | ND* |
| 5 | USA/NC58724-7/2024 | Turkey | Oropharyngeal swab | 17.9/B | - | - |
| 6 | USA/NC61042-GA/2024 | Turkey | Oropharyngeal swab | 18.3/B | - | - |
| 7 | USA/NC23734-GA/2024 | Turkey | Trachea | 18.6/B | + | - |
| 8 | USA/NC38371-GA/2024 | Turkey | Oropharyngeal swab | 19.1/B | - | - |
| 9 | USA/NC20487-GA/2024 | Turkey | Oropharyngeal swab | 19.7/B | + | - |
| 10 | USA/MI32566-GA/2024 | Turkey | Oropharyngeal swab | 19.9/B | - | - |
| 11 | USA/NC38172-GA/2024 | Turkey | Oropharyngeal swab | 19.9/B | - | ND |
| 12 | USA/OH42503-1/2024 | Turkey | Tracheal swab | 20.1/B | - | - |
| 13 | USA/MI31490-GA/2024 | Chicken | Oropharyngeal swab | 20.2/B | - | ND |
| 14 | USA/NC15504-GA/2024 | Turkey | Oropharyngeal swab | 20.3/B | - | - |
| 15 | USA/NC39727-GB/2024 | Turkey | Oropharyngeal swab | 21.1/B | + | + |
| 16 | USA/NC23734-GA/2024 | Turkey | Nasal turbinates | 21.1/B | - | - |
| 17 | USA/NC23018-GA/2024 | Turkey | Turbinates | 21.5/B | - | - |
| 18 | USA/OH35792-1/2024 | Turkey | Oropharyngeal swab | 21.7/B | - | - |
| 19 | USA/OH35792-2/2024 | Turkey | Oropharyngeal swab | 21.7/B | - | - |
| 20 | USA/NC18440-GA/2024 | Turkey | Trachea | 22.2/B | - | - |
| 21 | USA/MI30774-GA/2024 | Turkey | Oropharyngeal swab | 22.2/B | - | - |
| 22 | USA/NC23018/2024 | Turkey | Trachea | 22.3/B | - | - |
| 23 | USA/OH44164-1/2024 | Chicken | Tracheal swab | 22.5/A; 22.3/B | + (aMPV-A) | - |
| 24 | USA/OH38585/2024 | Turkey | Oropharyngeal swab | 22.7/B | - | - |
| 25 | USA/OH44441-1/2024 | Turkey | Tracheal swab | 23.0/B | - | - |
| 26 | USA/OH37369-1/2024 | Turkey | Oropharyngeal swab | 23.1/B | - | - |
| 27 | USA/IA21041-GB/2024 | Turkey | Oropharyngeal swab | 23.2/B | - | - |
| 28 | USA/OH37370-1/2024 | Turkey | Oropharyngeal swab | 23.5/B | - | - |
| 29 | USA/OH40821-1/2024 | Turkey | Oropharyngeal swab | 23.8/B | - | - |
| 30 | USA/OH38587-1/2024 | Turkey | Oropharyngeal swab | 24.4/B | - | - |
| 31 | USA/IA38478-GA/2024 | Turkey | Oropharyngeal swab | 24.5/B | - | - |
| 32 | USA/OH44162-1/2024 | Chicken | Tracheal swab | 24.7/B | - | - |
| 33 | USA/MI31490-GB/2024 | Chicken | Oropharyngeal swab | 24.8/B | - | ND |
| 34 | USA/OH37372-1/2024 | Turkey | Oropharyngeal swab | 25.3/B | - | - |
| 35 | USA/OH46642-1/2024 | Turkey | Tracheal swab | 23.9/A; 25.4/B | - | - |
| 36 | USA/NC39727-GA/2024 | Turkey | Oropharyngeal swab | 25.9/B | - | - |
| 37 | USA/IN36406/2024 | Turkey | Oropharyngeal swab | 27.9/B | - | - |
| 38 | USA/IN36405/2024 | Turkey | Oropharyngeal swab | 30.6/B | - | - |
| 39 | USA/OH37368/2024 | Turkey | Oropharyngeal swab | 30.7/B | - | - |
| 40 | USA/IN39902-2/2024 | Chicken | Oropharyngeal swab | 32.4/B | - | - |
| 41 | USA/OH36407/2024 | Turkey | Oropharyngeal swab | 33.0/B | - | - |
| 42 | USA/OH35686-1/2024 | Turkey | Oropharyngeal swab | 33.8/B | - | - |

*ND: Not Done. The samples that were aMPV-B VI-Positive are highlighted in grey color.

Supplemental Table S3. PCR testing on serial passages of selected aMPV-A and aMPV-B isolates in primary chicken embryo cells, with aMPV-A isolates in primary chicken embryo lung cells and aMPV-B isolates in primary chicken embryo fibroblast cells

| **Virus isolate** | **Subtype** | **Passage** | **aMPV-A PCR Ct** | **aMPV-B PCR Ct** | **aMPV-C PCR Ct** |
| --- | --- | --- | --- | --- | --- |
| USA/IA55601-6/2024 | A | P0 | 20.76 | ≥40 | ≥40 |
|  |  | P1 | 17.87 | ≥40 | ≥40 |
|  |  | P2 | 16.83 | ≥40 | ≥40 |
|  |  | P3 | 16.75 | ≥40 | ≥40 |
|  |  | P4 | 16.86 | ≥40 | ≥40 |
|  |  | P5 | 15.79 | ≥40 | ≥40 |
|  |  | P6 | 15.64 | ≥40 | ≥40 |
|  |  | P7 | 14.87 | ≥40 | ≥40 |
|  |  | P8 | 15.93 | ≥40 | ≥40 |
|  |  | P9 | 15.77 | ≥40 | ≥40 |
|  |  |  |  |  |  |
| USA/IA56509-5/2024 | A | P0 | 25.53 | ≥40 | ≥40 |
|  |  | P1 | 22.59 | ≥40 | ≥40 |
|  |  | P2 | 24.41 | ≥40 | ≥40 |
|  |  | P3 | 21.68 | ≥40 | ≥40 |
|  |  | P4 | 18.46 | ≥40 | ≥40 |
|  |  | P5 | 17.63 | ≥40 | ≥40 |
|  |  | P6 | 15.98 | ≥40 | ≥40 |
|  |  | P7 | 15.40 | ≥40 | ≥40 |
|  |  | P8 | 17.00 | ≥40 | ≥40 |
|  |  | P9 | 16.31 | ≥40 | ≥40 |
|  |  |  |  |  |  |
| USA/NC20487-GA/2024 | B | P0 | ≥40 | ≥40 | ≥40 |
|  |  | P1 | ≥40 | 36.18 | ≥40 |
|  |  | P2 | ≥40 | 20.66 | ≥40 |
|  |  | P3 | ≥40 | 16.89 | ≥40 |
|  |  | P4 | ≥40 | 15.24 | ≥40 |
|  |  | P5 | ≥40 | 13.14 | ≥40 |
|  |  | P6 | ≥40 | 12.65 | ≥40 |
|  |  | P7 | ≥40 | 11.00 | ≥40 |
|  |  | P8 | ≥40 | 11.55 | ≥40 |
|  |  | P9 | ≥40 | 11.71 | ≥40 |
|  |  |  |  |  |  |
| USA/NC23734-GA/2024 | B | P0 | ≥40 | 24.54 | ≥40 |
|  |  | P1 | ≥40 | 17.67 | ≥40 |
|  |  | P2 | ≥40 | 15.46 | ≥40 |
|  |  | P3 | ≥40 | 14.13 | ≥40 |
|  |  | P4 | ≥40 | 13.11 | ≥40 |
|  |  | P5 | ≥40 | 15.45 | ≥40 |
|  |  | P6 | ≥40 | 15.97 | ≥40 |
|  |  | P7 | ≥40 | 15.03 | ≥40 |
|  |  | P8 | ≥40 | 11.93 | ≥40 |
|  |  | P9 | ≥40 | 12.26 | ≥40 |

Supplemental Table S4. Avian metapneumovirus subtypes A and B included for sequence analysis in this study

| **Serial no.** | **Virus name** | **Subtype** | **Breed** | **Country** | **Collection year** | **GenBank accession no.** |
| --- | --- | --- | --- | --- | --- | --- |
| 1 | IT/Ty/A/259-01/03 | A | Turkey | Italy | 2003 | JF424833 |
| 2 | UK/LAH-A | A | Unknown | United Kingdom | Unknown | NC_039231 |
| 3 | UK/#8544 | A | Unknown | United Kingdom | Unknown | DQ666911 |
| 4 | CA/24-003048-001/2023 | A | Turkey | USA | 2023 | PP442012 |
| 5 | CA/24-003049-001/2023 | A | Turkey | USA | 2023 | PP442011 |
| 6 | MEX/3155/22 | A | Chicken | Mexico | 2022 | ON854014 |
| 7 | MEX/3154/22 | A | Chicken | Mexico | 2022 | ON854013 |
| 8 | MEX/3153/22 | A | Chicken | Mexico | 2022 | ON854012 |
| 9 | MEX/2390/20 | A | Chicken | Mexico | 2020 | ON854007 |
| 10 | MEX/2518/21 | A | Chicken | Mexico | 2021 | ON854006 |
| 11 | MEX/2759/21 | A | Chicken | Mexico | 2021 | ON854004 |
| 12 | MEX/2948/21 | A | Chicken | Mexico | 2021 | ON854003 |
| 13 | Brazil-SP/669/2003 | A | Chicken | Brazil | 2003 | MF093139 |
| 14 | NC/USA/ADRDL-6/2024 | B | Chicken | USA | 2024 | PP273461 |
| 15 | VA/USA/ADRDL-5/2024 | B | Turkey | USA | 2024 | PP273460 |
| 16 | VA/USA/ADRDL-4/2024 | B | Turkey | USA | 2024 | PP273459 |
| 17 | VA/USA/ADRDL-3/2024 | B | Turkey | USA | 2024 | PP273458 |
| 18 | NC/USA/ADRDL-1/2024 | B | Turkey | USA | 2024 | PP273456 |
| 19 | IA/SEP-RS1/2024 | B | Turkey | USA | 2024 | PQ382890 |
| 20 | SNU21004-E5/2021 | B | Chicken | South Korea | 2021 | OR461285 |
| 21 | SNU21004-V12/2021 | B | Chicken | South Korea | 2021 | OR461284 |
| 22 | SC1509_2000 | B | Chicken | South Korea | 2020 | OR461286 |
| 23 | 21004-PLQ7/2021 | B | Chicken | South Korea | 2021 | OM249787 |
| 24 | Korea/21004/2021 | B | Chicken | South Korea | 2021 | OM249786 |
| 25 | LN16-A/2023 | B | Chicken | China | 2023 | PP069785 |
| 26 | WH2022 | B | Unknown | China | 2022 | OP036743 |
| 27 | LN16/2016 | B | Chicken | China | 2016 | MH745147 |
| 28 | BR/1891/E2/19 | B | Unknown | Brazil | 2019 | OP572409 |
| 29 | BR/1890/E1/19 | B | Unknown | Brazil | 2019 | OP572408 |
| 30 | VCO3/60616 | B | Meleagris gallopavo | France | Unknown | AB548428 |
| 31 | Hungary/657/4/1989 | B | Turkey | Hungary | 1989 | MN729604 |
| 32 | Mallard/NL/1/2017 | C | Mallard | Netherlands | 2017 | OM256458 |
| 33 | MN/turkey/2a/97 | C | Turkey | USA | 2009 | FJ977568 |
| 34 | USA/15a/2001 | C | Goose | USA | 2001 | DQ009484 |
| 35 | Colorado_Vero | C | Unknown | USA | Unknown | AY590688 |
| 36 | USA-Colorado | C | Unknown | USA | Unknown | AY579780 |
| 37 | PL-2 | C | Pheasant | South Korea | Unknown | EF199772 |
| 38 | PL-1 | C | Pheasant | South Korea | Unknown | EF199771 |
| 39 | China/GDY/2011 | C | Muscovy duck | China | 2011 | KC915036 |
| 40 | China/2018/CL | C | Jinding duck | China | 2018 | PP662686 |
| 41 | China/2022/HF3 | C | Jinding duck | China | 2022 | OR365552 |
| 42 | China/2022/HL1 | C | Jinding duck | China | 2022 | OR365551 |
| 43 | China/S01/2011 | C | Duck | China | 2011 | KF364615 |
| 44 | NL/1/2019 | C | Mallard | Netherlands | 2019 | OM179883 |
| 45 | 1999/99178/France | C | Muscovy duck | France | 1999 | HG934338 |
| 46 | 1985/Fr85.1 | D | Turkey | France | 1985 | HG934339 |
| 47 | IA55601-6/2024_ PrimaryCells_P1 | A | Turkey | USA | 2024 | PV067035 |
| 48 | IA55601-6/2024_ PrimaryCells_P9 | A | Turkey | USA | 2024 | PV067036 |
| 49 | IA55601-6/2024_Vero_P4 | A | Turkey | USA | 2024 | PV067037 |
| 50 | IA55601-6/2024_Vero_P10 | A | Turkey | USA | 2024 | PV067038 |
| 51 | IA56509-5/2024_ PrimaryCells_P3 | A | Turkey | USA | 2024 | PV067039 |
| 52 | IA56509-5/2024_ PrimaryCells_P9 | A | Turkey | USA | 2024 | PV067040 |
| 53 | IA56509-5/2024_Vero_P4 | A | Turkey | USA | 2024 | PV067041 |
| 54 | IA56509-5/2024_Vero_P10 | A | Turkey | USA | 2024 | PV067042 |
| 55 | NC20487-GA/2024_ PrimaryCells_P3 | B | Turkey | USA | 2024 | PV067043 |
| 56 | NC20487-GA/2024_ PrimaryCells_P9 | B | Turkey | USA | 2024 | PV067044 |
| 57 | NC20487-GA/2024_Vero_P4 | B | Turkey | USA | 2024 | PV067045 |
| 58 | NC20487-GA/2024_Vero_P10 | B | Turkey | USA | 2024 | PV067046 |
| 59 | NC23734-GA/2024_ PrimaryCells_P1 | B | Turkey | USA | 2024 | PV067047 |
| 60 | NC23734-GA/2024_ PrimaryCells_P9 | B | Turkey | USA | 2024 | PV067048 |
| 61 | NC23734-GA/2024_Vero_P4 | B | Turkey | USA | 2024 | PV067049 |
| 62 | NC23734-GA/2024_Vero_P10 | B | Turkey | USA | 2024 | PV067050 |
